# Epirubicin carboplatin and 5-fluorouracil (ECarboF) chemotherapy in metastatic hormone refractory prostate cancer

**DOI:** 10.1038/sj.bjc.6602177

**Published:** 2004-09-21

**Authors:** A J Birtle, J C Newby, S J Harland

**Affiliations:** 1 The Meyerstein Institute of Oncology, The Middlesex Hospital, Mortimer Street, London W1N 8AA, UK

**Keywords:** combination chemotherapy, prostate cancer, ECarboF

## Abstract

The aim of this study was to examine the efficacy and toxicity of the epirubicin, carboplatin and 5-fluorouracil (ECarboF) regime in patients aged 70 or less with metastatic prostate cancer resistant to LHRH analogues. The majority of patients had previously received steroids as part of their systemic management and had progressive disease on steroids. In total, 80 patients were treated over a 6-year period, with objective response rates (PSA or radiological) of 45% and median time to relapse of 9.5 months. Median survival of the group was 9.2 months. In all, 32% of patients were alive at 12 months. Grade 3/4 neutropenia occurred in 34% of patients with an 8.7% rate of neutropenic sepsis. Grade 3/4 nonhaematological toxicity occurred in 28% of patients. For a substantial minority of patients with hormone refractory prostate cancer, combination chemotherapy can induce remission of significant duration. While similar responses have been documented for systemic cytotoxic–steroid combinations, the responses in this study are likely to reflect the activity of cytotoxic drugs alone.

The value of cytotoxic chemotherapy in patients with metastatic prostate cancer in progression following androgen suppression is uncertain. In recent times, mitozantrone has been shown to produce pain relief in a proportion of patients. In addition, using a fall in plasma prostate-specific antigen (PSA) as an end point, an additional 16–25% of patients (Berry *et al*, 2001; Kantoff *et al*, 2001) are seen to respond to the addition of mitozantrone to steroid in randomised studies, compared to the use of steroids alone (Tannock *et al*, 1996). A larger proportion of patients have been shown to respond to combined drug treatments in phase 2 studies. However, these combinations often comprise cytotoxic agents together with an oestrogen or a corticosteroid – the latter often given as prophylaxis against nausea or against the allergic manifestations of taxanes. Both oestrogens and corticosteroids have antitumour effects in prostate cancer (Fossa *et al*, 2001), and thus it is difficult to gauge the specific contribution of the cytotoxic drugs. Combination chemotherapy with 5-fluoruracil, epirubicin and cisplatin (ECF) in metastatic prostate cancer (Chao *et al*, 1997) showed an encouraging response rate – over 40% – in a limited number of patients. Carboplatin substitution for cisplatin (ECarboF) has been successfully implemented in the treatment of breast cancer (Bonnefoi *et al*, 1996), and reduces auditory and renal toxicity at the cost of additional myelotoxicity. We wished to establish the efficacy and tolerability of this therapy in a large group of patients with hormone-refractory metastatic prostate cancer, while ensuring that any benefit related to the cytotoxic agents rather than co-administered steroids.

## MATERIALS AND METHODS

### Eligibility

Patients with biopsy-proven adenocarcinoma of the prostate with recurrent or metastatic disease, which had failed first-line hormone therapy, were included in the study. Both symptomatic and asymptomatic patients were eligible. Those without symptoms had three successive rises in serum PSA, in keeping with the American Society of Therapeutic Radiation Oncology guidelines (Horwitz *et al*, 1998). Previous hormone therapy was continued, with the exception of nonsteroidal antiandrogens (see below) and a change of hormonal therapy was not permitted. All patients had a PSA greater than 20 ng ml^−1^, or bi-dimensionally measurable disease. In general, only patients <70 years, with WHO performance status 0–2, with no history of previous chemotherapy were eligible. No previous hemi body radiotherapy or intravenous strontium treatment was allowed. All patients were required to have white blood cell (WBC) count >3.0 × 10^9^ l^−1^ and platelets >100 × 10^9^ l^−1^. EDTA clearance was performed prior to commencing chemotherapy. Serum alkaline phosphatase and renal function was recorded prior to treatment and patients classified into prognostic subgroups (Fossa *et al*, 1992).

### Treatment

Patients received epirubicin 50 mg m^−2^ intravenously and carboplatin (calculated area under curve of 5) as an intravenous infusion, both 4 weekly. Initially, protracted venous infusion (PVI) 5-Fluorouracil (5FU) at a dose of 200 mg m^2^ day^−1^ was given via a Hickman line, but due to Hickman line difficulties, this was changed mid-way through the study to 450 mg m^−2^ as a 1 h infusion on days 1 and 15 with folinic acid 20 mg m^−2^. All patients with Hickman lines received low-dose anticoagulation, initially with 2 mg daily of warfarin. If the patient was taking endocrine therapy prior to chemotherapy, this was continued. If nonsteroidal antiandrogen therapy had been recently discontinued, 8 weeks were allowed to elapse before commencing chemotherapy to exclude any response due to androgen withdrawal. Patients who had responded to first-line hormonal therapy for <9 months were usually given chemotherapy on relapse, whereas those patients with longer response times were routinely given a trial of second-line hormonal therapy, including oral hydrocortisone, before chemotherapy was considered. Thus, the majority of patients had disease which was also progressive on hydrocortisone therapy. In these patients, dexamethasone as antiemetic prophylaxis was given. Otherwise, steroids were omitted from the antiemetic regime in view of their potential antitumour action. However, if nausea persisted despite 5-HT3 antagonists, oral and/or intravenous steroid therapy was considered. If there was a nadir platelet count of <50 × 10^9^ l^−1^ or pretreatment platelet count <100 × 10^9^ l^−1^, the carboplatin dose was reduced by 25%. If the nadir WBC was WHO grade 3 (neutrophils <0.5 × 10^9^ l^−1^), or pretreatment WBC was WHO grade 2 (2.0–2.9 × 10^9^ l^−1^), the epirubicin dose was reduced by 25%.

Diarrhoea and stomatitis of WHO grade 2 or above resulted in a 20% reduction of 5FU, or temporary cessation of treatment for those patients on PVI, followed by a 20% dose reduction. Six to eight cycles of treatment were given. WHO grade of toxicity was recorded at each cycle.

### Response criteria

Patients were assessed prior to each cycle of chemotherapy for symptomatic response or progression as described in the EORTC Phase III 30853 study of hormonal therapy in metastatic prostate cancer (Denis *et al*, 1993). Symptoms of progressive disease were defined as a progression of urological symptoms with the appearance of severe symptoms requiring surgical relief or catheterisation, weight loss of more than 10% within 1 year, or deterioration of WHO performance status. An increase in pain score as reflected by a step up in the WHO analgesia classification and ladder (Ventafridda *et al*, 1985) was also considered to reflect progression. However, objective measures of symptom scores such as Quality of Life tools and formal pain scores were not performed. Serum PSA levels were measured prior to each cycle. Commonly the PSA rose after chemotherapy commenced, prior to its falling and in some cases, a fall in PSA was seen as a late event. The overall PSA response to treatment was defined as a fall in the PSA levels to less than 50% of pretreatment values, maintained for at least 1 month after completion of six cycles of chemotherapy (‘study criteria’), a definition used in our previous study of ECF chemotherapy in the same patient group (Chao *et al*, 1997). PSA response was additionally defined using the PSA international consensus, as PSA level falling to less than 50% of pretreatment values and sustained for at least one further reading 4 weeks later (Bubley *et al*, 1999b). For measurable disease, complete response was defined as the complete resolution of disease sites and partial response as >50% reduction in the measurable lesions (measured in two directions) sustained for 1 month after completion. Progressive disease was defined as >25% increase in the size of measurable lesions or the appearance of new lesions. Stable disease was defined using the WHO definition of a less than 50% decrease in total tumour size and <25% increase in the size of the measurable lesions. Chemotherapy was discontinued if there was clinical or radiological deterioration by week 8. Otherwise, three cycles of chemotherapy were given before concluding that no response had been achieved. The decision to continue or stop chemotherapy was invariably made on the basis of patients' clinical progress, assisted by PSA values and occasionally radiological assessment. A rising PSA was based on a minimum of two successive increases. If chemotherapy appeared to be preventing progression, chemotherapy could be continued despite a stable PSA. Treatment was also stopped if there was patient intolerance or if six to eight cycles had been reached. If any steroid-naïve patient had been commenced on steroids because of vomiting or suspected spinal cord or nerve root compression, the response documented at the cycle immediately prior to starting steroids was used. This allowed an assessment of the response due to cytotoxic therapy alone to be recorded. Duration of response and survival were defined from the time chemotherapy was initiated.

### Sample size

The above regime is in regular use in our hospital. This report is generated at a time when the number of patients treated gives a lower confidence limit for response of greater than 25%.

## RESULTS

### Patient demographics (Table 1)

**Table 1:** Patient demographics

Number of patients	80
*Age (years)*	
Median	64
Range	48–72
	
*ECOG performance status*	
0	43
1	39
2	8
Symptomatic	63 (78%)
Asymptomatic	17
	
*Site of disease*	
Bone only	56
Bone+soft tissue	20
Local recurrence only	4
Prostate-specific antigen on entry (ng ml^−1^)	196
Range	4.7–1832
	
*Prognostic groups at entry*^a^	
Group 1	24
Group 2	49
Group 3	7

a Prognostic subgroups according to Fossa *et al* (1992).

Between December 1995 and March 2002, 80 patients were entered, 73 of whom had elevated PSA levels (greater than 20 ng ml^−1^) prior to treatment. In all seven patients with nonelevated PSA, disease was measurable on liver ultrasound and/or chest CT. The median age was 64 years (range 48–72 years) with four patients above the upper age limit, all of whom were of performance status 0–1 and hence entered at the physician's discretion. Patients were classified into prognostic groups according to the Fossa classification (Fossa *et al*, 1992), wherein duration of hormone response, performance status, serum creatinine and alkaline phosphatase are predictive of survival. Subgroups 1 2 and 3 were defined, with predicted prognoses of 10, 6 and 3 months respectively. The majority of patients selected for this study were thus in relatively good prognostic categories. In total, 63 out of 80 patients (78%) had symptoms of progressive disease as previously described. Patients received a median of five cycles (range 1–8 cycles) and dose reductions or delays were seen in 46 patients (57%). A total of 59 out of 80 patients (73%) had disease that was progressive on hydrocortisone therapy, with a further 21 patients who had not received steroids as antitumour systemic therapy. Seven of these 21 steroid-naive patients received dexamethasone during the study due to persistent vomiting (five patients) or neurological deterioration (two patients).

### Toxicity

A total of 75 patients (93%) were evaluable for toxicity (Table 2

**Table 2:** Grade 3/4 Haematological and nonhaematological toxicities in 75 evaluable patients

Haemoglobin	13 (16%)
Neutrophils	27 (33.8%)
Platelets	13 (16%)
Neutropenic sepsis	7 (8.7%)
Hickman line thrombus/re-insertion/shoulder pain	7/45 (15%)
Renal	1
Skin	2
Emesis	8(of which five were in steroid-naïve group)
Diarrhoea	2
Stomatitis	4
Infection	2
Cardiac	1
Haematuria	2

). The rate of neutropenic sepsis was low (8.7%) with nonhaematological WHO Grade 3 or 4 toxicity occurring in 22 patients (27.5%). One patient developed left ventricular failure (New York Heart Classification Grade 4) shortly after completing the sixth cycle of chemotherapy, but his cardiac function subsequently improved. There was one deep vein thrombosis despite prophylactic low-dose warfarin anticoagulation, and one patient suffered a subdural haematoma, the aetiology of which may have been related to his anticoagulation or to an unrelated intracranial haemorrhage. One patient also experienced rectal bleeding which required discontinuation of chemotherapy.

### Tumour response (Table 3)

**Table 3:** Response to EcarboF chemotherapy (80 patients evaluable)

*PSA response*	
Defined per International criteria^a^	42/73 (57%)
Defined per study criteria^b^	30/73 (41%)
	
Radiological response^c^	6/7
*Total objective responses*	
Using International PSA response criteria	48/80 (60%)
Using study PSA response criteria	36/80 (45%)
	
Symptomatic response (doctor's assessment)	41/63^d^

a Defined as >50% fall in initial level maintained for at least 4 weeks.

b Defined as >50% fall in initial level maintained for at least 4 weeks from completion of chemotherapy.

c PSA not elevated prior to treatment.

d In the 63 patients who were symptomatic at the initiation of treatment.

In all, 36 out of the 80 (45%) patients responded by PSA (study criteria) and/or radiological criteria (95% confidence interval (CI) 29–60%) with a median time to first suppression of PSA of 7 weeks (range 3–14 weeks) from commencing chemotherapy. When the specific study criteria of PSA response, defined as the fall in PSA maintained for 1-month postcompletion of chemotherapy was used, responses were seen in 30 out of 73 (41%) patients. When PSA response as defined by International Criteria was used, PSA responses were seen in 42 of the 73 (57%) patients with an elevated PSA prior to treatment. In all, 12 patients did not maintain the fall in PSA after completion of treatment. A slowing of the rate of rise of serum PSA accompanied by a symptomatic response as previously described was seen in a further five patients. Symptomatic response was seen in 41 out of 63 (65%) of patients who had been symptomatic prior to starting treatment. Of the seven patients with a pretreatment PSA of <20 ng ml^−1^, radiological responses, two of which were complete responses, were seen in six patients. The median duration of response was 9.5 months (range 7–17 months). Median survival was 9.2 months (range 1–29 months). In total, 40% of patients were alive at 12 months and 11% at 24 months.

## DISCUSSION

Chemotherapy in prostate cancer has been traditionally discounted, after disappointing results in the 1980s. Indeed, the combination of mitozantrone and prednisolone (Berry *et al*, 2002) although showing improvement in symptoms showed no survival benefit. This finding was reflected in previous trials of chemotherapy in prostate cancer comparing chemotherapy with best supportive care alone. Poor response rates of 10–15% to single agents with a median duration of response of 6–9 months have been quoted (Brausi *et al*, 1995). However, early studies in the pre-PSA era often relied on acid phosphatase levels and patient symptoms as markers of response, as only 20% of patients with metastatic prostate cancer, have measurable soft tissue disease (Figg *et al*, 1996). Post-therapy PSA levels have become widely accepted as a surrogate end point in the evaluation of treatment responses in hormone-refractory prostate cancer (Smith *et al*, 1998). One suggestion is that a decline of 50% or greater from baseline sustained over 2 months reflects a response (Kelly *et al*, 1993). We have used the Prostate Specific Antigen Working Group (Bubley *et al*, 1999a) criteria of a greater than 50% fall in serum PSA from pretreatment levels, sustained for at least 4 weeks but also our own specific definition of the PSA fall being maintained for a month or greater after completion of six cycles of chemotherapy. Previous work from this institution has shown response rates of 40% using epirubicin, cisplatin and 5-flurouracil (Chao *et al*, 1997). However, significant toxicity is encountered with ECF, particularly nausea, renal toxicity, peripheral neuropathy and ototoxicity (Ross *et al*, 2002). Carboplatin substitution for cisplatin minimises renal, auditory and neurological toxicity and simplifies the out patient regime but may prove more myelotoxic. It has not been used previously in metastatic prostatic tumours. In other Phase II studies, the combination of estramustine and docetaxel (Petrylak, 2002) has PSA response rates of up to 74 with a 77% 1-year survival. However, neutropenia rates were significant (43%) and prednisolone was also given at high dose as part of the premedication regime with each cycle. It is not clear, therefore, the extent to which docetaxel contributes to the high response rate in the steroid–cytotoxic combination.

**Figure 1:**
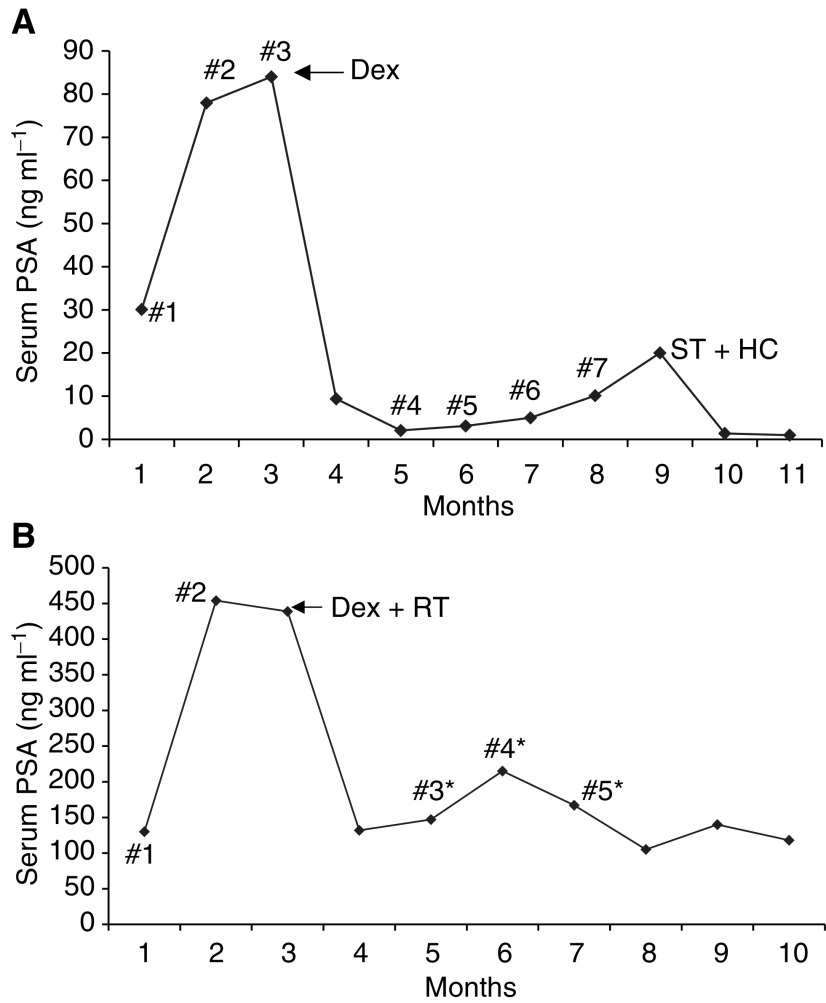
(**A**) PSA trend in response to chemotherapy and addition of steroids in Patient 1. 1–7: chemotherapy cycles; Dex: dexamethasone commenced for a period of 6 weeks; ST; stilboestrol; HC: hydrocorticosone. (**B**) PSA trend in response to chemotherapy and subsequent radiotherapy and steroids in Patient 2. 1–5: chemotherapy cycles; Dex+RT: dexamethasone and radiotherapy commenced; ^*^dexamethasone given as antiemetic.

We have previously presented data on the first 34 patients treated with the ECarboF regime (Newby *et al*, 1999). The response rate of ECarboF is similar (45 *vs* 43%) to that seen in a study from this institution with ECF (Chao *et al*, 1997). In this previous study, there was a 19% rate of Hickman line thrombosis, which has been reduced to 6% (three out of 45 patients) by low-dose warfarin anticoagulation. Other Hickman line problems including shoulder pain and lines becoming dislodged occurred in four patients. These problems lead to the discontinuation of 5-FU as a PVI and the use of the 1 h infusion of 5-FU on days 1 and 15. Previous endocrine therapy was continued to ensure that any tumour clone that was still hormone responsive did not relapse. Unlike other current regimes where high response rates have been seen, a change in hormonal management in addition to chemotherapy was not permitted, and thus this study reflects the response to chemotherapy alone. In those patients where clinical symptoms necessitated commencement of steroids, assessment of tumour response was documented prior to starting dexamethasone. Although some authors have documented no extra antitumour benefit from dexamethasone at high doses in combination with taxane and estramustine containing chemotherapy regimes (Weitzman *et al*, 2000), the antitumour effect of steroids alone has been well documented, giving better subjective responses in terms of improvements in pain score and performance status, and similar PSA responses to antiandrogen therapy (Fossa *et al*, 2001). The beneficial but confounding effects of steroids on tumour response have been demonstrated in two steroid-naïve patients treated in our institution with ECarboF subsequent to the current series (Figure 1A and B

). The administration of dexamethasone for spinal cord compression in these two patients was followed by a prompt fall in PSA, which may not have been seen with chemotherapy alone.

This study confirms the efficacy and manageable toxicity of the ECarboF regime in selected patients with hormone refractory prostate cancer receiving treatment as outpatients. The number of dose reductions and treatment delays illustrate that this is an aggressive regime. It would, therefore, not be suitable for the majority of patients, but should be considered for the sizeable minority in whom combination chemotherapy is appropriate. The encouraging response rate from ECarboF implies that combination chemotherapy is superior to single-agent cytotoxic treatments. This conclusion would require a randomised study, however, where factors such as quality of life and overall survival would additionally need to be compared.
